# Ebola Virus NP Binding to Host Protein Phosphatase-1 Regulates Capsid Formation

**DOI:** 10.21203/rs.3.rs-2963943/v1

**Published:** 2023-06-06

**Authors:** Asrar Ahmad, Bersabeh Tigabu, Andrey Ivanov, Marina Jerebtsova, Tatiana Ammosova, Palaniappan Ramanathan, Namita Kumari, Christine A. Brantner, Colette A. Pietzsch, Ghadeer Abdullah, Anastas Popratiloff, Steve Widen, Alexander Bukreyev, Sergei Nekhai

**Affiliations:** Howard University; The University of Texas Medical Branch at Galveston; Howard University; Howard University; Howard University; UTMB: The University of Texas Medical Branch at Galveston; Howard University; GWU: George Washington University; UTMB: The University of Texas Medical Branch at Galveston; Howard University; GWU: George Washington University; UTMB: The University of Texas Medical Branch at Galveston; UTMB: The University of Texas Medical Branch at Galveston; Howard University

**Keywords:** Ebola virus, nucleocapsid, nuclear protein, protein phosphatase-1, 1E7–03, small molecule EBOV inhibitor, split NanoBiT, Transmission electron microscopy

## Abstract

The Ebola virus (EBOV) transcriptional regulation involves host protein phosphatases PP1 and PP2A, which dephosphorylate the transcriptional cofactor of EBOV polymerase VP30. The 1E7–03 compound, which targets PP1, induces VP30 phosphorylation and inhibits EBOV infection. This study aimed to investigate the role of PP1 in EBOV replication. When EBOV-infected cells were continuously treated with 1E7–03, the NP E619K mutation was selected. This mutation moderately reduced EBOV minigenome transcription, which was restored by the treatment with 1E7–03. Formation of EBOV capsids, when NP was co-expressed with VP24 and VP35, was impaired with NPE 619K. Treatment with 1E7–03 restored capsid formation by NP E619K mutation, but inhibited capsids formed by WT NP. The dimerization of NP E619K, tested in a split NanoBiT assay, was significantly decreased (~ 15-fold) compared to WT NP. NP E619K bound more efficiently to PP1 (~ 3-fold) but not B56 subunit of PP2A or VP30. Cross-linking and co-immunoprecipitation experiments showed fewer monomers and dimers for NP E619K which were increased with 1E7–03 treatment. NP E619K showed increased co-localization with PP1α compared to WT NP. Mutations of potential PP1 binding sites and NP deletions disrupted its interaction with PP1. Collectively, our findings suggest that PP1 binding to the NP regulates NP dimerization and capsid formation, and that NP E619K mutation, which has the enhanced PP1 binding, disrupts these processes. Our results point to a new role for PP1 in EBOV replication in which NP binding to PP1 may facilitate viral transcription by delaying capsid formation and EBOV replication.

## INTRODUCTION

The Ebola virus (EBOV) is a non-segmented, negative strand RNA virus responsible for a deadly human disease [1]. The 2013–2016 outbreak in West Africa was the most severe on record, with 28,000 cases and 11,000 deaths [2]. The most recent outbreak in the Democratic Republic of the Congo in 2018–2020 resulted in over 3,400 confirmed cases and 2,280 deaths [3], followed by additional outbreaks and cases [4]. Consequently, it is essential to create new drugs and treatments against EBOV.

The EBOV genome comprises seven genes encoding eight proteins: nucleoprotein (NP), polymerase cofactor (VP35), matrix protein (VP40), glycoprotein (GP) and secreted glycoprotein sGP, transcriptional activator (VP30), membrane-associated protein (VP24) and RNA-dependent RNA polymerase (L) [5]. We previously found that inhibiting the host protein phosphatase-1 (PP1) induces VP30 phosphorylation, thus shifting the balance of transcription-replication activity of the EBOV polymerase complex toward replication [6]. Recently, it was discovered that NP recruits the host protein phosphatase PP2A-B56, which dephosphorylates the VP30 N-terminal serine cluster, resulting in an upregulation of viral transcription [7]. A subsequent study showed that serine-arginine protein kinase 1 (SPRK1) interacts with the R_26_xxS_29_ motif of VP30 and phosphorylates VP30 Ser-29 [8].

PP1 and PP2A belong to the phosphoprotein phosphatase (PPP) superfamily. The PP1 holoenzyme is composed of a catalytic subunit (PP1α, PP1β or PP1γ) and a regulatory PP1-interacting protein that directs PP1 to a specific location in the cell and determines its activity and substrate specificity [9]. Over 200 validated PP1 interactors bind to the PP1 catalytic subunits using multiple binding motifs such as RVxF, SpiDoc, SILK, MyPhoNE, ΦΦ and NIPP1-helix [10, 11]. Small molecules that can compete with these docking motifs can be used to block the binding of the interactors to PP1, thus functionally disrupting distinct PP1 holoenzymes and inhibiting PP1-mediated processes, such as the replication of EBOV by changing the phosphorylation of viral or host proteins.

Given the prevalence of the RVxF-type docking motif among 95% of PP1 interactors [12], we developed a series of small molecules to target the RVxF-motif binding pocket in silico and identified compounds 1H4 [13] and 1E7–03 [14]. *In vivo*, 1E7–03 was effective against HIV-1 [15], and reduced LPS-induced lung inflammation in HIV-1 transgenic mice [16, 17]. We tested 1E7–03 against EBOV and showed that it induced VP30 phosphorylation and effectively suppressed EBOV infection in cell cultures [6]. Moreover, we found that 1E7–03 inhibits Marburg virus [18], Rift Valley fever virus [19] and Venezuelan Equine Encephalitis Virus [20]. Treatment with 1E7–03 shifted the transcription/replication balance of the EBOV polymerase complex towards replication [6]. However, 1E7–03 quickly degraded in mice [15], and its degradation products had no suppressive effects against EBOV [21]. We identified various analogs of 1E7–03, such as C31 and 1E7–07, that were stable in mice or mouse serum, but were about 10 times less effective in EBOV inhibition than 1E7–03 [21, 22]. Thus, 1E7–03 remains the best PP1-targeting inhibitor of EBOV to date.

In the current study, we analyzed the effects of 1E7–03 on EBOV replication and virus mutation during a continuous long-term virus culture. We observed a single mutation, NP E619K, after passage 4 in viruses purified from 1E7–03 treated cultures. To explore the involvement of PP1 in EBOV replication further, we examined the effects of this mutation on viral replication/transcription, viral capsid formation, interaction of NP with PP1 and PP2A, and on NP dimerization. Analysis using transmission electron microscope (TEM) showed that while Wild-type (WT) NP efficiently formed helical capsids with VP35 and VP24, no capsids were detected for the NP E619K mutant. However, NP E619 was able to form capsids in 1E7–03 treated cells, while capsid formation by the WT NP was inhibited by 1E7–03. We used the split NanoBiT system to assess the dimerization of NP and its binding to PP1, PP2A, and VP30. The NP E619K mutant dimerized with less efficiency than the WT NP, yet it bound PP1 with greater affinity than WT NP. By contrast, the NP E619K mutant bound to PP2A and VP30 with similar intensity. To investigate whether NP E619K can oligomerize/polymerize and the effect of 1E7–03, we conducted DPS-crosslinking experiments that revealed fewer monomers and dimers in the soluble fraction for the NP E619K mutant. Treatment with 1E7–03 increased both monomers and dimers for NP E619K, but not for the WT NP. We employed fluorescent microscopy to determine co-localization of PP1 with NP in live cells using NP-RFP and PP1-eGFP fusions. Split Nano-Bit analysis of PP1 binding to the mutated NP or NP with various deletions, suggested the presence of potential PP1 binding sites in NP. Collectively, our study suggests that enhanced PP1 binding to NP could reduce NP polymerization and disrupt capsid formation, implying that PP1 might regulate capsid formation, potentially facilitating EBOV transcription.

## RESULTS

### EBOV adaption to 1E7–03 treatment

**Figure 1:**
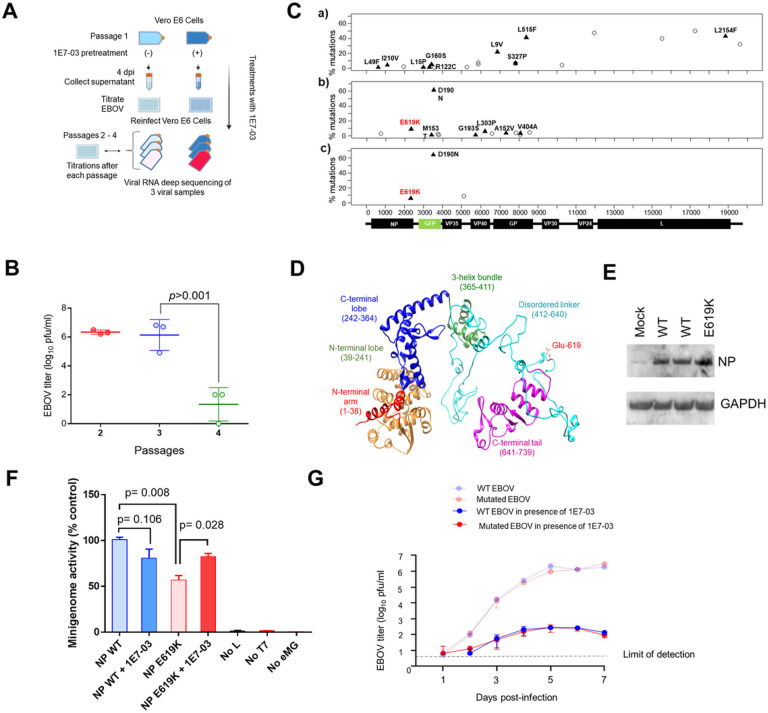
EBOV adaptation to 1E7–03. (A) Workflow showing long-term 1E7–03 treatment strategy of EBOV infected Vero E6 cells. Vero-E6 cell monolayers were infected with EBOV-eGFP, cultured with or without 1E7–03, and supernatants were collected at 10–11 days post infection and used for reinfection. (B) EBOV replication from the virus collected after the indicated passages. Mean ± SD viral titers based on triplicate samples. (C) Triplicate viral RNA samples from passage 4 were deep sequenced. The frequencies of the identified mutations across the EBOV genome are shown (a,b,c). Missense mutations are shown as shaded triangles, and silent mutations are shown as open circles. Schematics of the EBOV-eGFP genome showing only the open reading frames of individual viral genes and eGFP are shown. The E619K mutation in the NP gene detected in two of the three replicates is highlighted in red. (D) The NP structure prepared from the *de novo* model and reconstituted in Chimera 1.14 showing NP structured domains as well as NP E619K mutation. (E) NP WT and NP E619K subcloned into the pCEZ vector were expressed in Vero-E6 cells. NP protein expression was analyzed by Western blot with anti-NP antibodies, and GAPDH antibodies used as a loading control. (F) EBOV transcription activity of NP E619K mutant determined by minigenome assay with either WT NP or NP E619K mutant. Where indicated, the cells were also treated with 1E7–03. At 48 hrs post-transfection, luciferase activity was measured. Data are the mean ± SD of triplicates. (G) Kinetics of EBOV replication with NP E619K mutation. EBOV titers were measured for the virus collected at 1-, 3-, 5- and 7-days post infection. Data are means ± SD of triplicates.

We had previously demonstrated that PP1-targeting compounds 1E7–03 and its derivatives C31 and 1E7–07 were effective at inhibiting replication of EBOV [6, 21, 22]. To further investigate the mechanism of 1E7–03-mediated inhibition of EBOV replication, we conducted a continuous viral culture study (Fig. 1A for workflow). First, Vero-E6 cells were pre-treated with 3 μM 1E7–03 for 24 hrs, and then infected with a recombinant EBOV that expresses eGFP (EBOV-eGFP) [23] at a multiplicity of infection of 0.01 PFU/cell. The cells were treated with 3 μM 1E7–03 every 24 hrs for 4 days (Supplemental Fig. 1). After 4 days, the supernatants were collected, virus was titrated, and used in the following round of infection. As the viral titer from the first passage was low, the maximum available amount (15 PFU) was used to infect the cells in the next passage. Passage 2 was performed in the same way, except that the treatment was extended to 11 days (Supplemental Fig. 2). At 11 days post infection, the supernatants were collected, titrated, and used to infect the monolayers for passage 3. The third passage began with a MOI 0.01 PFU/cell and was continued for 11 days with daily 1E7–03 treatments (Supplemental Fig. 3). Over the course of 10 days, the treatment was repeated. On day 11, the virus titers from passages 2 and 3 were nearly equal, while the titer from passage 4 was lower. (4.8 log_10_ reduction, Fig. 1B). Following passage 4, three viral samples were isolated from both 1E7–03 treated and untreated cultures. Deep sequencing of the viral RNA was performed to identify mutations present in the viral genome of the samples from the treated 1E7–03 cultures. Subsequently, several mutations in these proteins were found (Supplemental Table 1). In contrast to the treated cultures, the samples obtained from the untreated ones only exhibited mutations in the VP24 protein (Supplemental Table 2). These mutations coincided with VP24 alterations seen in the treated cultures. When the three samples were compared, the NP E619K mutation (Fig. 1C) had the most significant p-value in sample 2 and was the only single mutation observed in sample 3. Therefore, we focused our subsequent analysis on the NP E619K mutation.

The NP E619K mutation is located within the unstructured linker that connects the N-terminal structured lobes and the C-terminal tail of NP [24] (Fig. 1D). To investigate whether NP E619K mutation had an effect on NP expression, it was introduced in the NP expression vector and the expression was analyzed in Vero-E6 cells. Immunoblotting analysis showed similar expressions of both the WT NP and NP E619K (Fig. 1E). We then evaluated the impact of the NP E619K mutation on EBOV replication/transcription using an EBOV minigenome system (Fig. 1F). Vero-E6 cells were cultured in 24-well plates and transfected with the EBOV minigenome and plasmids expressing components of the EBOV polymerase complex (L, VP35, and VP30) under the control of T7 polymerase and a T7 polymerase-expressing vector [6]. Additionally, WT NP or NP E619K mutants were expressed from co-transfected plasmids with CMV promoter controlling the expression. Renilla luciferase activity was measured 48 hrs after transfection. When compared with the WT NP, the mutation resulted in a less than two-fold decrease in the minigenome reporter gene signal (Fig. 1F), indicating that NP E619K was functional in EBOV transcription. The reintroduction of the NP E619K mutation to EBOV-eGFP caused a slight delay in replication kinetics at day 5 post infection compared to WT EBOV-eGFP. However, 1E7–03 still effectively inhibited both viruses (Fig. 1G and Supplemental Fig. 4), indicating that the adaptation was insufficient to fully overcome the inhibition.

### The NP E619K mutation impairs EBOV capsid formation but facilitates capsid formation in cells treated with 1E7–03

**Figure 2:**
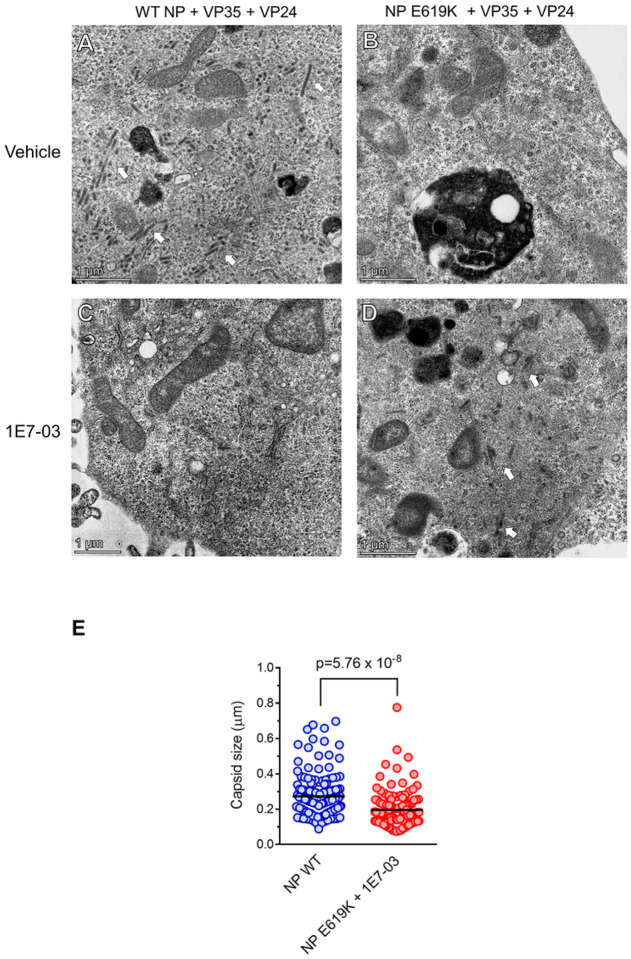
EBOV capsid formation by NP E619K is facilitated by 1E7–03. HEK293 cells were co-transfected with vectors expressing NP-RFP, VP24 and VP35. Cells were fixed, stained, and sectioned as described in Materials and Methods. Imaging was performed on a Talos 200X transmission electron microscope. (A-D) Electron microscopy of HEK 293 cells expressing WT NP, VP24 and VP35 (panel A) and treated with 1E7–03 (panel C), or NP E619K mutant, VP24 and VP35 (panel B) and treated with 1E7–03 (panel D). The bars indicate 1 μm scale. Arrows indicate capsid formation. (E) Liner size of capsids was measured in Image J.

Using transmission electron microscopy (TEM), we tested the effect of NP mutation on EBOV capsid formation. We expressed either WT NP or an NP E619K mutant fused with RFP in HEK 293 cells along with VP35 and VP24 proteins. The WT NP was able to facilitate capsid structures formation (Fig. 2A). In contrast, no capsid formation was observed when the NP E619K mutant was used (Fig. 2B). Flow cytometry analysis revealed similar expression levels of WT NP and NP E619K (Supplemental Fig. 5), indicating that the NP E619K mutant might be deficient in capsid formation. To assess the effect of 1E7–03 treatment on capsid formation, TEM images of HEK 293 cells expressing NP, VP35 and VP24 were analyzed after treatment with DMSO vehicle or 1E7–03. While capsids were absent in 1E7–03-treated cells expressing WT NP (Fig. 2C), capsids were observed in cells expressing the NP E619K mutant and treated with 1E7–03 (Fig. 2D). We found that the linear size of EBOV capsids in cells expressing the NP E619K mutant and treated with 1E7–03 was 1.4-fold smaller than that of the capsids observed in the cells expressing WT NP (Fig. 2E). These results suggest that the NP mutation facilitates E619K the virus adaptation to 1E7–03 treatment and promotes capsid formation under the drug treatment.

### NP E619K mutation prevents NP dimerization and increases binding of PP1

**Figure 3:**
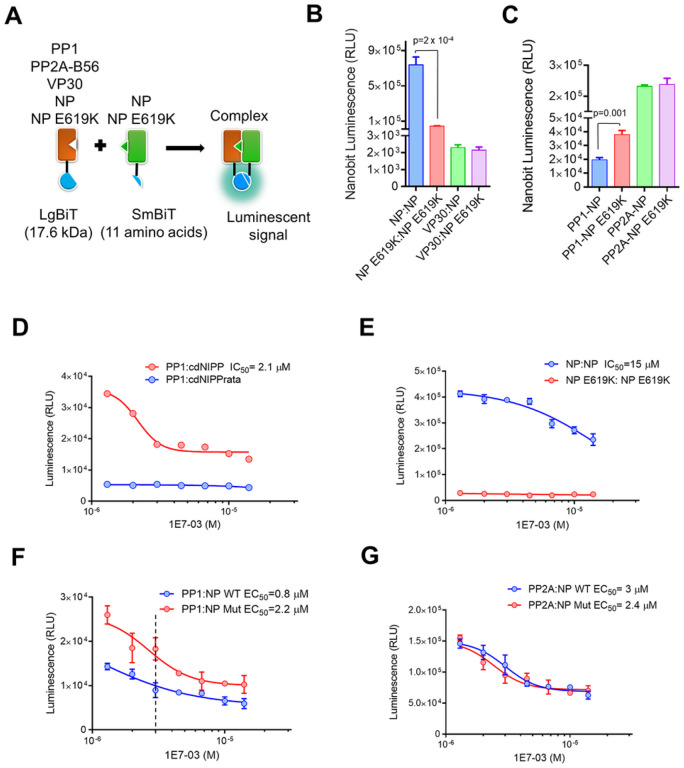
EBOV NP E619K mutation deregulates NP-NP interaction and increases PP1 binding. (A) A schematic representation of the split NanoBiT system, as well as the PP1 and NP constructions that were utilized. PP1, PP2A-B56, VP30, NP and NP E619K were fused at the C-terminus of large Bit (LgBiT). NP or NP E619K was tagged at the N-terminus with small Bit (SmBit). Reconstitution of LgBit and SmBit produced luminescent signal in the presence of cell-permeable substrate furimazine. (B) NP E619K mutation reduced NP-NP interaction. LgBiT- and SmBit-tagged genes were co-transfected into HEK293T cells. NanoBiT luminescence activity was measured at 30 hrs post transfection. Each value represents the mean ± SD from three independent experiments. (C) NP E619K mutation increases NP-PP1 interaction but had no effect on NP-PP2A-B56 interaction. NanoBit complementation assay was carried out as in panel B and data are shown as the mean ± SD from three independent experiments. (D) 1E7–03 inhibits prevents PP1:cdNIPP1 interaction. HEK293T cells were co-transfected by NanoBiT-tagged genes for PP1 and cdNIPP1, or PP1 and cdNIPP1 RATA mutant (described in Materials and methods) for 24 hrs. The cells were then treated with different concentrations (1.3–14 μM) of 1E7–03 for an additional 6 hrs and NanoBiT luminescence activity was measured. Data are the mean ± SD of triplicates. (E) 1E7–03 treatment reduces NP-NP interaction. Cells were co-transfected with NP:NP and NP E619K:NP E619K and the experiment was carried out as described in panel A. Data are shown as mean ± SD. (F-G) Effect of 1E7–03 on PP1-NP and PP2A-B56-NP interactions. Cells were co-transfected with the indicated NanoBiT vectors and treated with 1E7–03. NanoBiT luminescence activity was measured and plotted as mean ± SD of triplicates.

Recently we have used a Split NanoBiT-based system for studying the interaction of PP1 with its regulatory partners [22]. By fusing PP1 to the large bit (LgBit) and PP1 binding peptides (such as the central domain of NIPP1 (cdNIPP1)) to the small bit (SmBit), we were able to assess the effect of the NP E619K mutation on NP dimerization and PP1 binding (see Materials and Methods for vector details) (Fig. 3A). WT NP formed dimers efficiently, as indicated by the strong NanoBit signal for the NP-NP interaction (Fig. 3B). In contrast, the self-interaction of the NP E619K mutant was 1,650-fold lower than that of WT NP, suggesting a decrease in dimerization (Fig. 3B). The binding of VP30 to both WT NP and the NP E619K mutant was similar (Fig. 3B). However, the NP E619K bound PP1 three-fold more strongly than the WT NP (Fig. 3C). Both WT and NP E619K mutant bound equally well PP2A B56 subunit (Fig. 3C). Taken together, these results suggest that the NP E619K mutant was impaired in dimerization, but had increased binding to PP1, suggesting that PP1 binding to NP might negatively affect NP dimerization.

Next, we tested the effect of 1E7–03 on NP-NP and NP-PP1 interaction. We first assayed the effect of 1E7–03 on PP1 binding to its known interactor, cdNIPP1, and compared it to cdNIPP1 RVxF mutant (cdNIPP1rata, V201A/F203A mutation) that was deficient in PP1 binding as indicated by the reduced NanoBiT signal (70-fold reduction, Fig. 3D). For this analysis, 293T cells were co-transfected with the specified NanoBiT plasmids for 24 hrs and then treated for 6 hrs with serial dilutions of 1E7–03 (1.3–14 μM). We found that 1E7–03 disrupted the PP1-cdNIPP1 binding (IC_50_ = 2.1 μM) (Fig. 3D), and also affected NP-NP interaction to a lesser extent (IC_50_ = 15 μM) (Fig. 3E). This suggests that 1E7–03 may not have a direct effect on NP homodimerization. As was previously mentioned, the interaction between NP E619K-NP E619K was weaker than that of WT NP-WT NP, and 1E7–03 had no effect on this weak interaction (Fig. 3E). However, 1E7–03 was able to disrupt both PP1-NP interaction (IC_50_ = 0.8 μM) and the PP1-NP E619K interaction (IC_50_ = 2.2 μM) (Fig. 3F). As PP1-NP E691K binding was stronger, the quantity of PP1 bound to NP E619K mutant even in the cells treated with 3 μM 1E7–03 was higher than that of untreated NP-PP1 (Fig. 3F, dashed line). This indicates that the NP E619K mutation allowed for adequate PP1 binding even under the presence of 3 μM 1E7–03. Additionally, 1E7–03 had an equal suppressing effect on the interactions between WT NP and NP E619K with the PP2A B56 subunit (Fig. 3G). Split NanoBiT system experiments revealed that both PP1 and NP were co-expressed equally well (Supplemental Fig. 6A). 1E7–03 treatment had no effects on PP1 expression, but slightly reduced NP expression (Supplemental Fig. 6B and C). This suggests that the effects of 1E7–03 were not due to the significant reduction of PP1 or NP protein expression.

**Figure 4:**
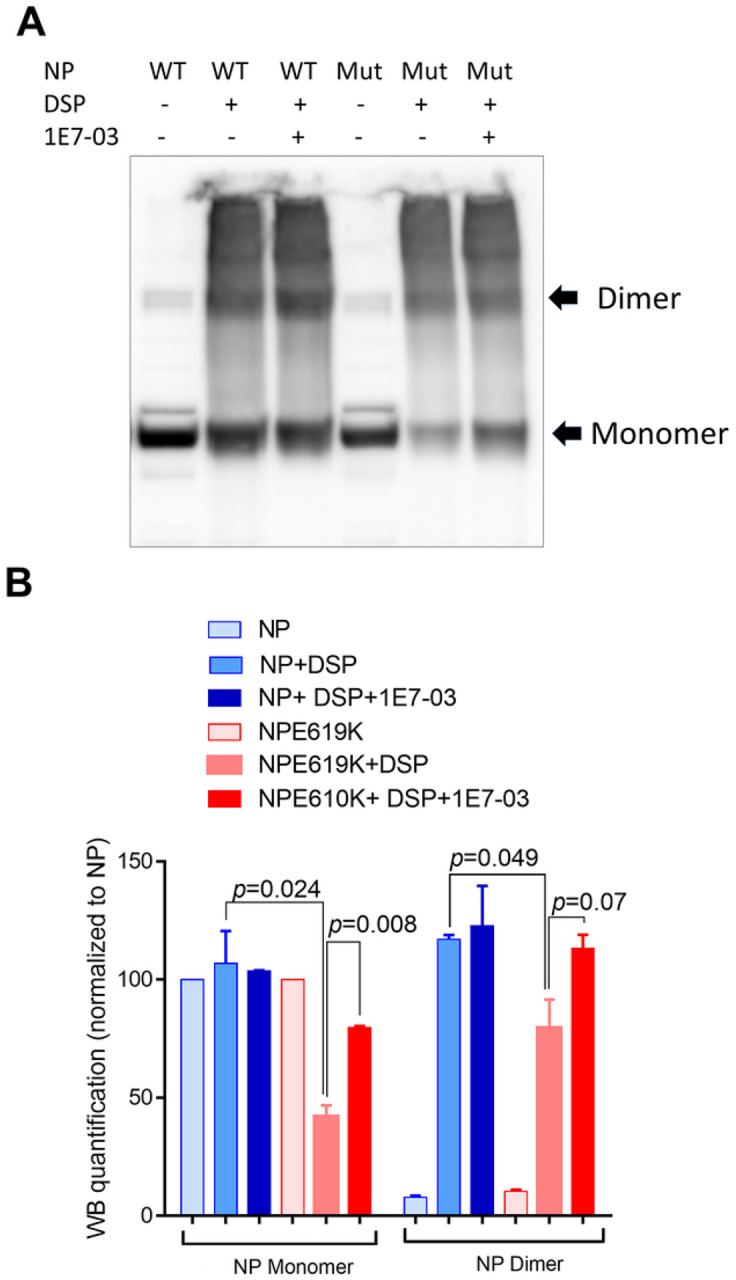
NP dimerization analyzed with DSP cross linking. 293 cells were transfected with vectors expressing NP WT or NP E619K, and then either left untreated or treated with 1E7–03 for 24 hrs. At 48 hrs post transfection, the cells were treated with 2 mM DSP for 45 minutes. The cell lysates were resolved on SDS PAGE under non-reducing conditions. Monomers and dimers are indicated by arrows. (B) Quantification of three independent experiments from panel A.

To validate the NanoBiT results, we next analyzed the effect of 1E7–03 treatment on the oligomerization of the WT NP and the NP E619K mutant using dithiobis[succinimydylpropionate] (DSP) crosslinking followed by non-reducing SDS gel electrophoresis [25]. Crosslinking with DSP allowed us to detect both monomers and dimers of the WT NP and NP E619K. After DSP cross-linking, WT NP formed diners and higher oligomeric forms compared to non-DSP-treated WT NP (Fig. 4A). In contrast, DSP cross-linking of NP E619K resulted in the formation of significantly fewer monomers and dimers compared to WT NP, despite having equal amounts of monomers the input not treated with DPS (Fig. 4A and B). This suggests that the NP E619K mutant is compromised in its ability to form oligomers. Treatment with 1E7–03 significantly increased the amount of NP E619K monomers in DSP-treated non-reduced samples and also had a trend toward increasing the amount of dimers (Fig. 4A and B). In contrast, 1E7–03 treatment had no effect on the amount of NP WT monomers and dimers (Fig. 4A and B). These observations suggest that the inability of NP E619K to form dimers might be due to the sequestration of the mutant NP within an insoluble complex within the cells. This could reduce the amount of soluble NP E619K monomers available for oligomerization. Treatment with 1E7–03 seems to release the mutant NP into the soluble fraction, thus facilitating its oligomerization.

To further confirm the interaction between NP and PP1, we analyzed the co-precipitation of NP with PP1. 293T cells were co-transfected with vectors expressing V5-tagged PP1α and Flag-tagged NP. The cells were treated with either vehicle (DMSO) or 10 μM 1E7–03. PP1α was immunoprecipitated with anti-V5 antibodies, resolved on 10% SDS polyacrylamide gel and immunoblotted with anti-Flag antibodies to detect NP protein and anti-V5 antibodies to detect PP1 (Supplemental Fig. 7). NP E619K showed a slightly higher affinity for PP1 (Supplemental Fig. 7B). Treatment with 1E7–03 reduced PP1 binding to the WT NP, whereas PP1 binding was increased with NP E619K (Supplemental Fig. 7).

Overall, these observations indicate that the NP E619K mutation is defective in dimerization and has a stronger binding affinity to PP1 compared to the WT NP.

### NP co-localizes with PP1α

**Figure 5:**
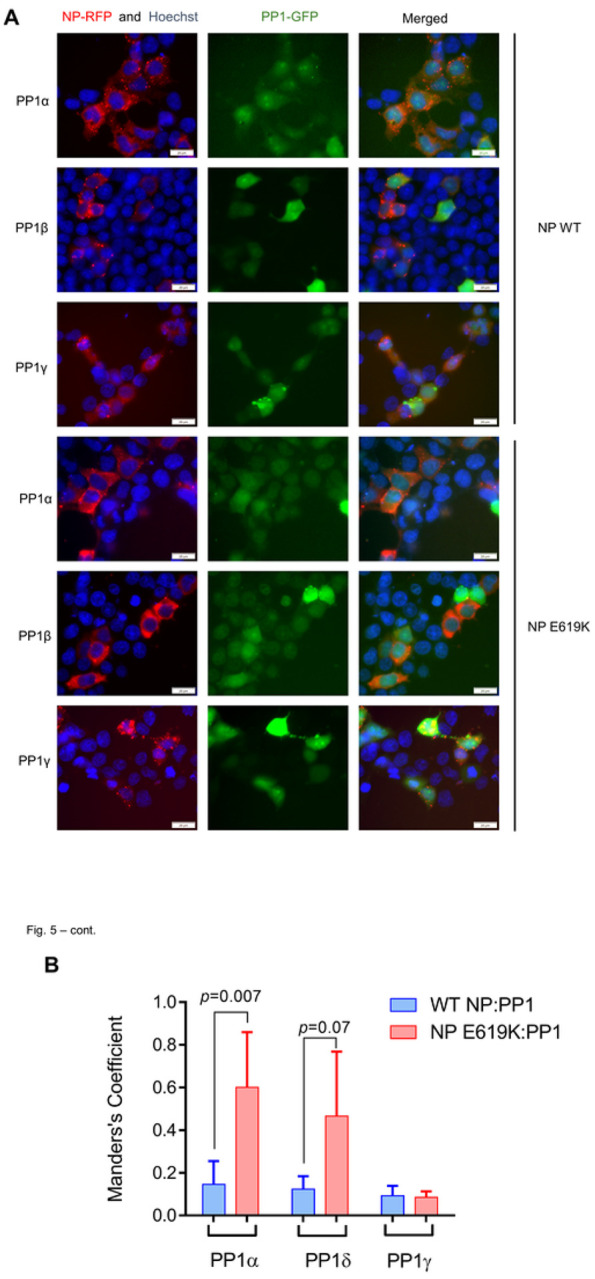
PP1 co-localized with NP. (A) Fluorescent imaging of PP1 and NP co-localization. 293 cells were transfected with vectors expressing NP-mCherry and PP1(α, β/δ or γ)-GFP as indicated in Materials and Methods. At 48 hrs post transfection, the cells were stained with Hoechst 33342 and imaged at 600x magnification using filters for Texas Red, FITC and DAPI on Olympus IX73. (B) Quantification of PP1 and NP colocalization. The colocalization was assessed in Image J using the JACoP plug-in, which allows Pearson’s correlation analysis. Prior to the correlation analysis, the image colors were split, and the threshold parameters were adjusted.

To determine which PP1 isoform might interact with NP in cultured cells, we examined the co-localization of NP with PP1 by utilizing live-cell fluorescence imaging of co-expressed NP-RFP and PP1-eGFP fusion proteins (Fig. 5A). We observed robust co-localization of NP with PP1α, but not with PP1 β/δ or PP1γ (Fig. 5A). We then calculated Mander’s coefficient to validate and differentiate the co-localization of WT and mutant NP. There was a significant increase in the co-localization of the NP E619K mutant with PP1α, as well as an inclination towards increased co-localization with PP1 β/δ, but no change in co-localization with PP1γ when compared to WT NP (Fig. 5B). Thus, NP is likely to bind PP1α.

### Analysis of PP1 binding sites on NP

**Figure 6:**
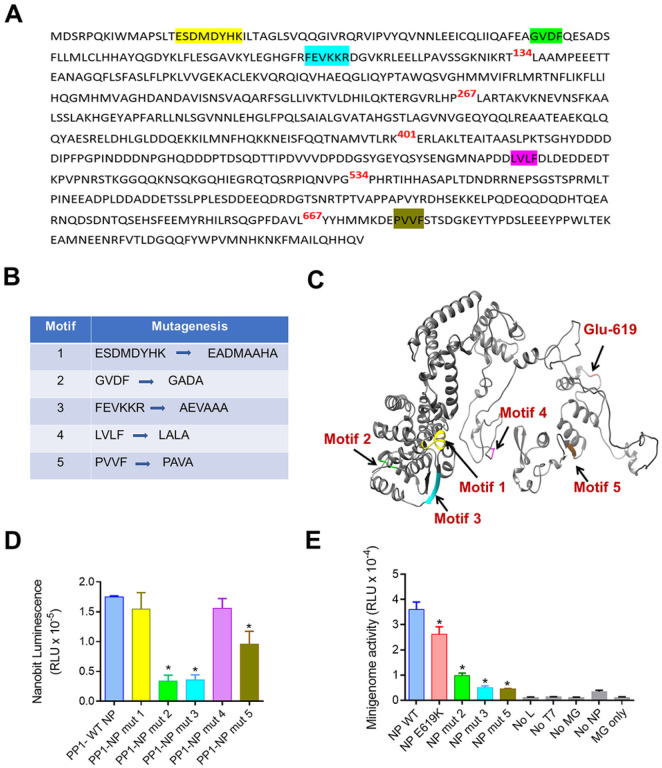
Potential PP1 binding sites on NP and their effects on the interaction with PP1. (A) Potential PP1 binding motifs in the NP amino acid sequence are indicated in yellow (motif 1), green (motif 2), magenta (motif 3), purple (motif 4) and forest green (motif 5). (B) Changes in the potential PP1 binding motifs introduced by site-directed mutagenesis. (C) NP de novo structure constructed in Chimera 1.14, showing location of potential PP1-binding motifs. (D). LgBiT- and SmBit-tagged genes were co-transfected into HEK293T cells, and NanoBiT luminescence was measured at 24 hrs post transfection. Each value represents the mean ± SD from three independent experiments. *p<0.01. (E) Effect of mutation in NP’s PP1 binding sites on EBOV minigenome. HEK293T cells were transfected with the components of EBOV minigenome system except NP which was expressed from a plasmid under the control of CMV promoter. Forty-eight hrs post-transfection luciferase activity was measured. Data are the mean ± SD of triplicates. *p<0.001.

To identify PP1 binding sites on NP, we analyzed the presence of potential PP1 binding motifs within the NP sequence and found five potential binding sites (Fig. 6A). To evaluate their functionality, we mutated them (Fig. 6B) and tested their effect of mutations on PP1-NP interaction in the split NanoBit system (Fig. 6C). Motifs 1, 2 and 3 are located within the N-terminal NP core (Fig. 6C), while motifs 4 and 5 are located within the disorder linker and the C-terminal tail domain, respectively (Fig. 6C). Mutations of motifs 2 and 3 had a strong negative effect on PP1-NP interaction (Fig. 6D), while mutation of motif 5 showed an intermediate effect (Fig. 6D). In contrast, mutations in motifs 1 and 4 had no adverse effect on PP1-NP interaction (Fig. 6D). We further tested these mutations in the EBOV minigenome and found that the mutations in either motif 2, 3, or 5 significantly impacted EBOV transcription (Fig. 6E).

**Figure 7:**
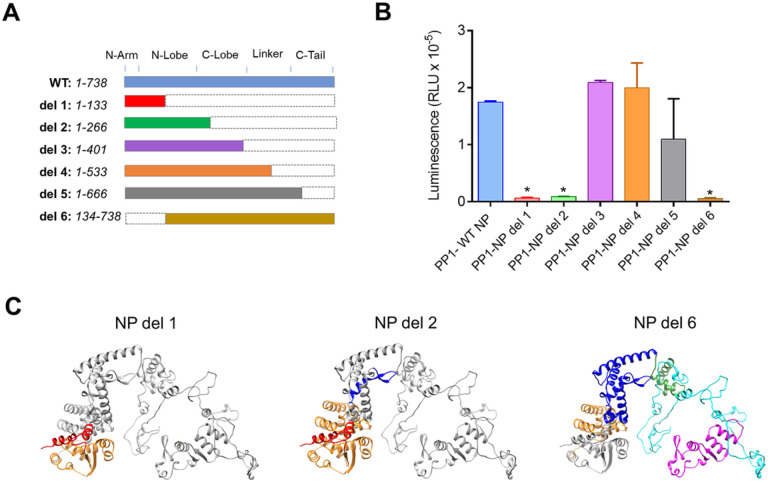
Effects of NP deletion mutants on NP-PP1 interaction. (A) NP deletion mutants generated to test NP binding to PP1 are represented schematically. (B) Effect of NP deletions on PP1 binding. PP1 LgBiT- and NP deletions SmBit-tagged genes were co-transfected into HEK293T cells and NanoBiT luminescence was measured at 24 hrs post transfection. Each value represents the mean ± SD from three independent experiments. *p<0.01. (C) NP *de novo* structures prepared from de novo reconstituted NP structure in Chimera 1.14 showing NP deletion mutants 1,2 and 6.

We next analyzed the effect of NP deletions on PP1 binding. We generated six NP deletion mutants that spanned the entire NP sequence (Fig. 7A). NP deletion mutants 1 and 2, which contained only part of the N-terminal domain (1–133 aa and 1–266 aa, respectively), did not bind PP1 (Fig. 7B). NP lacking the first 133 amino acids also did not bind PP1 (Fig. 7B). NP deletion mutants containing 1–401 amino acids or 1–533 amino acids could bind PP1 to the level of WT NP (Fig. 7B). The NP mutant containing 1–666 amino acids also retained the ability to bind PP1. Structures of the NP deletion mutants 1, 2 and 6, which were unable to bind PP1, are shown in Fig. 7C. It is likely that these deletions interfere with the interaction of NP and RNA, suggesting that the NP-PP1 interaction may involve RNA binding.

Taken together, our data indicate that PP1 interacts with NP, and that the E619K mutation in NP enhances this interaction, while decreasing NP-NP binding and compromising EBOV capsid formation but facilitating NP capsid formation in the presence of 1E7–03.

## DISCUSSION

In this study, we extended our previous analysis of the role of PP1 in EBOV replication by investigating the long-term effects of 1E7–03 treatment in viral culture. We identified the NP E619K mutation that developed during the long-term treatment of EBOV infected Vero-E6 cells. We observed that, although this mutation had only a mild effect on EBOV transcription resulting in less than 50% decrease in minigenome replication, it might still facilitate EBOV capsid formation when cells expressing NP E619K were treated with 1E7–03. Our analysis of viral capsid assembly indicated that there was a striking difference between the WT NP and the NP E619K mutant. In cells that expressed WT NP, VP35, and VP24, we observed the formation of viral capsids, whereas no capsids were detected in the cells that expressing NP E619K mutant, VP35 and VP24. Furthermore, no capsids were found in WT NP, VP35 and VP24 - expressing cells after treatment with 1E7–03, while capsids were observed in NP E619K, VP35 and VP24 expressing cells treated with 1E7–03, although the capsid were shorter in size. This observation suggests that EBOV might have adapted to 1E7–03 by being able to form capsids under drug pressure. To obtain further insight into the effect of the NP E619K mutation, we analyzed NP dimerization, as well as NP E619K binding to PP1, PP2A B56 and VP30, using split NanoBiT system and crosslinking experiments. We found that the NP E619K mutation impaired NP’s ability to dimerize, reducing dimerization efficiency by more than 1,000-fold. NP E619K mutant bound to PP1 more efficiently but had the same binding of PP2 B56 and VP30 as the WT NP. Analysis of the effect of 1E7–03 in the split NanoBiT system showed that at 3 μM 1E7–03 concentration, which was used in viral passaging experiments, NP E619K mutant retained PP1 binding comparable to untreated WT NP-PP1. Crosslinking with DSP showed that the NP E619K mutant had reduced amounts of monomers and dimers compared to WT NP. Remarkably, treatment with 1E7–03 increased both monomers and dimers, suggesting that PP1 may aid in sequestration of NP, which hinders its ability to dimerize. Co-precipitation of PP1α with NP demonstrated that the NP E619K mutant binds more PP1 and that this enhanced binding was preserved in the presence of 1E7–03. On the contrary, WT NP binds less PP1 and its weaker binding was also sustained even in the cells treated with 1E7–03. Together, these results support the idea that NP E619K is more efficient at inducing capsid formation than WT NP in the presence of 1E7–03. Furthermore, NP was found to co-localize with PP1α, with the colocalization being particularly pronounced for NP E619K. This agrees with the findings from the split NanoBiT system, which showed a stronger interaction between NP E619K and PP1. Our analysis of PP1’s interaction with NP revealed at least three potential PP1 binding sites. However, deletion analyses highlighted the importance of an intact N-terminal domain for the binding of PP1 to NP, suggesting RNA binding might be involved. Consequently, PP1 might bind to NP indirectly through a PP1 regulatory subunit capable of binding RNA. As PP1 interacting regulatory proteins include more than 200 validated members [10, 11], it is possible that one of these subunits with an RNA binding capability is involved in this interaction. Further analysis with the use of techniques such as proximity labelling is required to identify this subunit and to better understand PP1 binding.

**Figure 8:**
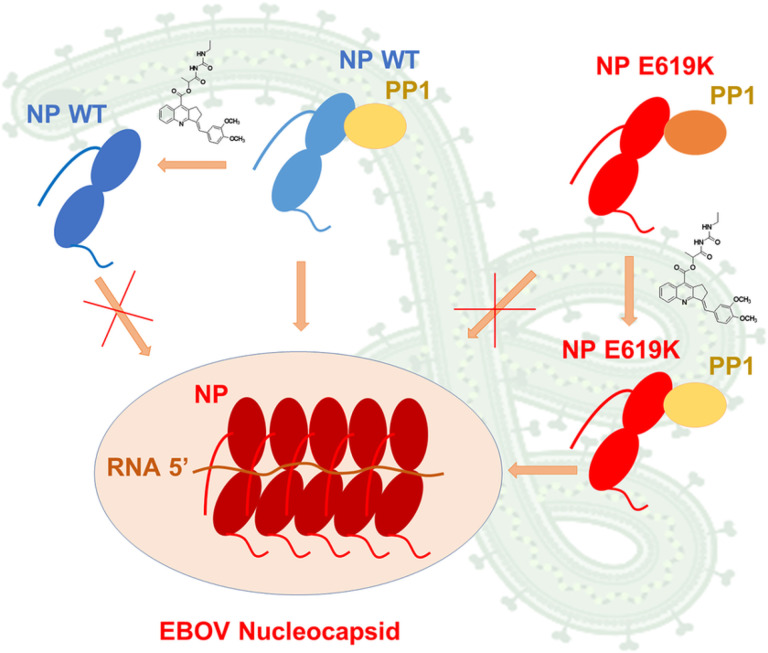
Model of NP-PP1 interaction and the effect of 1E7–03 on capsid formation. NP binding to PP1 leads to NP dimerization and EBOV nucleocapsid formation. The addition of 1E7–03 prevents PP1 binding to WT NP and blocks nucleocapsid formation. The enhanced association of NP E619K with PP1 prevents its dimerization and capsid formation. Treatment with 1E7–03, dissociates the excess of PP1 and allows NP E619K to dimerize and form nucleocapsids.

Our study points to the hitherto unrecognized role of PP1 in regulating NP capsid assembly (summarized in Fig. 8). We propose that NP binds PP1 and this binding is requisite for NP oligomerization and subsequent capsid formation. Treatment with 1E7–03 precludes PP1 binding with WT NP and thus blocks nucleocapsid formation. The increased PP1 binding by NP E619K seems to impede oligomerization. Treatment with the PP1-targeting 1E7–03 compound allows partial PP1 dissociation from NP E619K mutation and thus induces capsid formation. In accord with the previous study [7], we observed strong NP interaction with PP2A, which was not affected by NP E619K mutation. We hypothesize that NP might interact with both PP2A and PP1, and that PP1 and PP2A might work in concert. An example of concert recruitment and fine-tuning of PP1 and PP2A can be seen in RepoMan, a protein encoded by *CDCA2*, which acts as a scaffold for both PP1 and PP2A B56 [26, 27]. Phosphorylation of RepoMan by Aurora B on Ser-893 and Thr-394 prevents PP1 binding [27, 28]. Conversely, phosphorylation of RepoMan by CDK1 on Ser-591 promotes the recruitment of PP2A B56, which reverses Ser-893 phosphorylation and enables re-recruitment of PP1 and dephosphorylation of M phase proteins [29–31]. In this regard, the NP E619K mutation, which introduces a positive charge instead of a negative charge, may mimic NP dephosphorylation which would facilitate PP1 recruitment. It is thought that the function of PP2A which bound to NP may include dephosphorylation of an unknown NP residue(s) to aid PP1 recruitment, prevent capsid formation and induce transcription. The N-terminal part of NP (first 450 aa) is necessary for NP-NP interaction and, as well as the following 150 amino acid residues, is critical for nucleocapsid formation and viral replication [32]. Our previous global phosphoproteomic analysis of EBOV virions identified twenty NP phosphorylation sites [33]. Fourteen of these sites (Thr-536, Ser-541, Thr-545, Thr-563, Thr-597, Ser-598, Thr-601, Thr-603, Tyr-686, Thr-687, Tyr-688, Ser-691, Tyr-696 and Thr-701) were found within the large unstructured sequence that connects the N-terminal and C-terminal domains of NP [33]. Previous studies identified phosphorylation of Thr-563, Ser-581, Ser-587 and Ser-647 in NP expressed in cultured cells [34]. As existing crystal structures of EBOV NP only capture parts of the N-terminal region [35–37], and a C-terminal domain [38], we previously constructed a full-length model of NP using *de novo* prediction, which showed highly stable globular-like ‘structured’ sections during an equilibrium MD simulation in a periodic water box [33]. The NP residues 412–645 were highly flexible during MD simulation [33], suggesting that these residues are accessible for PP1 or PP1 regulatory subunit binding. We also identified ten phosphorylation sites on VP35 that was packaged in EBOV virions [33]. Interestingly, dephosphorylation of Thr-210 blocked EBOV transcription and prevented VP35 binding to NP [33], suggesting that NP-associated phosphatases can also control EBOV transcription by dephosphorylating VP35.

The NP E619K mutation resides near the VP30-binding PPxPxY motif that was also found in the host RBBP6 protein [39]. Recently, a mass spectrometry approach identified additional PPxPxY-containing host proteins, including hnRNP L, hnRNPUL1, and PEG10 that all strongly interact with VP30 [40]. While we did not observe any changes in VP30 binding to NP E619K, which was extremely weak in our split NanoBiT system, it is possible that NP E619K mutation affects the VP30 exchange between host proteins and NP. Further analysis is needed to test the binding of NP E619K with host proteins and other factors such as VP35.

Taken together, our findings suggest that host PP1 has a unique role that involves interacting with NP and controlling NP dimerization and EBOV capsid formation.

## MATERIALS AND METHODS

### Chemicals and reagents

1E7–03 (purity above 98%) was synthesized by Enamine (Kyiv, Ukraine) as previously described [14]. Dimethyl sulfoxide (DMSO), acetone, hydrochloric acid and sodium hydroxide were obtained from Fisher Scientific (Fair Lawn, NJ). Sodium acetate (pH 5.2) was from Quality Biological (Gaithersburg, MD). Phosphate buffered saline (pH 7.4) was from Life Technologies (Grand Island, NY).

### Cells and media

Vero-E6 and HEK293T cells were purchased from the American Type Culture Collection (Manassas, VA). Vero-E6 cells were cultured in modified Eagle medium (Life Technologies) with 10% fetal bovine serum (FBS) and 1% gentamicin (Life Technologies). The 293T cells were cultured in Dulbecco’s modified Eagle’s medium (Invitrogen, Waltham, MA) containing 10% FBS and 1% antibiotic solution (penicillin and streptomycin).

### Experiments with infectious EBOV

All experiments using infectious EBOV were performed under the Biosafety Level 4 (BSL-4) containment of the Galveston National Laboratory. Vero-E6 cell monolayers were grown in 12 or 24-well plates and treated for 1 hr at 37°C, 5% CO_2_ with compounds diluted in maintenance MEM medium (Life Technologies) containing 2% FBS (Hyclone, Logan, UT), 0.1% gentamicin sulfate (Corning, Corning, NY), 1% Nonessential Amino Acids (Sigma), and 1% sodium pyruvate (Sigma). Cell monolayers were infected with the recombinant EBOV that expresses eGFP (EBOV-eGFP) from an added gene for 1 hr at 37°C [23]. After adsorption, monolayers were washed three times with phosphate buffered saline (PBS), a fresh compound in the maintenance medium was added to each well, and monolayers were incubated at 37°C for 4 days (passage 1), 11 days (passages 2 and 3) or 10 days (passage 4) post-infection.

To quantify EBOV-eGFP titers in Vero-E6 cell supernatants, confluent Vero-E6 monolayers were inoculated with 10-fold serially diluted supernatants, adsorbed for 1 hour at 37°C, 5% CO_2_ and subsequently coated with an overlay of the medium containing 0.9% methylcellulose (Sigma, St. Louis, MO). Fluorescent viral plaques were counted after 4 days using a UV microscope.

### EBOV sequencing

Triplicate Vero-E6 cell monolayers were treated with 0.3 μM 1E7–03 for 24 hrs and then infected with recombinant EBOV WT virus expressing eGFP at a MOI 0.01 PFU/cell. The treatment was repeated every 24 hrs for 4–11 days. Supernatants were collected and used for titration and infection during the next round of selection passage. The supernatants from prior passages were used to infect new Vero-E6 cell monolayers. The passages were repeated four times. At the end of each selection cycle, RNA was extracted from cell monolayers and were lysed in 1 ml of TRIzol (Thermo Fisher Scientific, Waltham, MA) for 10 min at room temperature before the samples were removed from the BSL-4 containment. RNA isolated from cell monolayers after the 4th passage was sequenced. Quantification and purity of resuspended total RNA were determined by NanoDrop (Thermo Fisher Scientific). Approximately 1–4 μg of RNA was used to prepare libraries using the Ribo-Zero Stranded kit (Illumina, Hayward, CA) according to the manufacturer’s recommendations, and 50 base paired end reads were sequenced on the HiSeq1500 sequencer.

Raw reads (50 bp) were mapped using the EBOV reference sequence (NCBI Reference Sequence: NC_002549.1) using BWA mem (version 0.7.15, http://bio-bwa.sourceforge.net/). Visualization of the reads across this reference was accomplished with the Integrative Genomics Viewer (http://software.broadinstitute.org/software/igv/) [41].

### Mini-genome system

Minigenome was assembled as previously described, using pCEZ-NP, pCEZ-VP35, pCEZ-VP30, pCEZ-L and pC-T7 plasmids kindly provided by Dr. Yoshihiro Kawaoka. Vero-E6 cells were co-transfected with the minigenome plasmids using Mirus transfection reagent (Mirus Bio, Madison, WI). Transcription was measured at 48 hrs post-transfection by the luciferase assay (Promega, Madison, WI) and it was normalized to viable cell number.

### Plasmids

#### PP1 and cdNIPP1 expression plasmids.

Plasmids expressing PP1α, PP1β, and PP1γ fused to eGFP, cdNIPP1 (Residues 140–225)-eGFP fusion and cdNIPP1 RATA (V201A and F203A) mutant fused to eGFP were kindly provided by Mathieu Bollen (KU Leuven, Belgium).

#### NP expression plasmids.

The glutamic acid in position 619 of NP was substituted to lysine by overlap extension PCR using Pfx polymerase (Thermo Fisher Scientific) with two sets of external primers flanking 5’ and 3’ ends of pCEZ-NP sequence and two internal primers containing the mismatched bases. After sequence confirmation, the mutated plasmids were digested with MfeI and NheI enzymes and cloned back into pCEZ-NP vector. WT NP or NP E619K mutated plasmid were transfected in Vero-E6 cells using TransIT-LT1 Transfection Reagent (Mirus) at 3 μl per μg of plasmid DNA, and cell lysates were harvested 48 hrs post-transfection to confirm expression by western blotting. Blots were stained with Rabbit anti-EBOV NP Antibody (IBT Bioservices, Rockville, MD) and GAPDH (14C10) Rabbit mAb (Cell Signaling, Danvers, MA).

To prepare WT NP-mCherry and NP E619K-mCherry expression vectors, pcDNA3.1(-) plasmid was digested with Not1 and Kpn1 restriction enzymes and purified on the agarose gel. The mCherry fragment was amplified by PCR from pcDNA3.1-mCherry plasmid with forward CATGGCAATCCTGCAACATCATCAGAAGggcgaggaggataacatggccatc and reverse GTTTAAACTTAAGCTTGGTACCTACTTGTACAGCTCGTCCATGCCGCCGGTGG primers. PCR fragments of WT NP and NP E619K were amplified with forward AACGGGCCCTCTAGACTCGAGCGGCCGCATGGATTCTCGTCCTCAGAAAATCTGG and reverse GATGGCCATGTTATCCTCCTCGCCCTTCTGATGATGTTGCAGGATTGCCATG primers from pCEZ NP and pCEZ NP E619K expression vectors described above. All fragments were combined in one vector with Gibson assembly kit (New England BioLab, Ipswich, MA; cat# E2611S) according to the manufacturer’s protocol.

#### NanoBiT vectors.

We evaluated all combinations of expression constructs to determine the best combination and orientation for fusions of tested proteins to LgBiT and SmBiT, as recommended by Promega. To simplify multiple cloning procedures, the entry pF4A CMV vector was designed with SgfII and PmeI cloning sites. To clone the DNA fragments of all tested proteins, their sequences were PCR amplified with primers linked to SgfII and PmeI cloning sites. The WT NP and NP E619K mutant expression plasmids described above were amplified with forward GCGATCGCCATGGATTCTCGTCCTCAGAAAATCTGG and reverse GTTTAAACCTGATGATGTTGCAGGATTGCC primers. PP1 was amplified with GCGATCGCCATGTCCGACAGCGAGAAGCTCAACC and GTTTAAACGAATTCGAGCTCGGTA primers. The cdNIPP1 and cdNIPP1 RATA were amplified with the same GCGATCGCCATGGGTGGAGAGGATGATGAAC and GTTTAAACGAATTCGAGCTCGGTA primers because they have identical 5’ and 3’ ends. EBOV VP30 expression plasmid developed in Dr. Bukreev’s lab was amplified with forward ACGACTCACTATAGGGCTAGCGATCGCCATGGAAGCTTCATATGAGAG and reverse GTACCGAGCTCGAATTCGTTTAAACAGGGGTACCCTCATCAGACCATGAGC primers. PP2 B56 clone was obtained from GenScript and amplified with forward GACTCACTATAGGGCTAGCGATCGCCATGTCGTCGTCGTCGCCGCCGGCGG and reverse TAGAGGATCCCCGGGTACCGAGCTCGAATTCGTTTAAACTTCGGCACTTGTATTGCTGAG primers.

Mutagenesis of potential PP1 binding motifs in the NP protein was carried out in pcDNA3.1(-) NP plasmid using the primers listed below. Mutation of sequence ESDMDYHK to EADMAAHA was carried out with forward GAGTCTCACTGAAGCTGACATGGCTGCCCACGCGATCTTGACAGCAG and reverse CTGCTGTCAAGATCGCGTGGGCAGCCATGTCAGCTTCAGTGAGACTC primers. Mutation of sequence GVDF to GADA was carried out with forward CAGGCCTTTGAAGCAGGTGCCGATGCTCAAGAGAGTGCGGAC and reverse GTCCGCACTCTCTTGAGCATCGGCACCTGCTTCAAAGGCCTG primers.

Mutation of sequence FEVKKR to AEVAAA was carried out with forward GAAGGGCACGGGTTCCGTGCTGAAGTCGCGGCGGCTGATGGAGTGAAG and reverse CTTCACTCCATCAGCCGCCGCGACTTCAGCACGGAACCCGTGCCCTTC primers. Mutation of sequence mutated LVLF to LALA was carried out with forward GCACCAGATGACTTGGCCCTAGCCGATCTAGACGAGGAC and reverse GTCCTCGTCTAGATCGGCTAGGGCCAAGTCATCTGGTGC primers. Mutation of sequence the forward GATGAAGGATGAGCCTGCTGTTGCCAGTACCAGTGATG and reverse CATCACTGGTACTGGCAACAGCAGGCTCATCCTTCATC primers were used to mutate the sequence PVVF to PAVA. Further cloning of mutated NP was conducted exactly as described above. To facilitate exchange NP deletion mutants cloning, we created SgfII and PmeI cloning sites in pcDNA3.1(-) vector. Then PCR NP amplified deletion products were cloned directly in the pF4A CMV entry vector. NP deletion mutant 134–738 aa was produced with forward ACGGGCCCTCTAGACTCGAGCGGCCGCGATCGCCATGGATTCTCGTCC and reverse TTTAAACTTAAGCTTGGTACCGTTTAAACTGTTCTCTTAATGTTTTTTCC primers. The forward ACGGGCCCTCTAGACTCGAGCGGCCGCGATCGCCATGGATTCTCGTCC and reverse TTTAAACTTAAGCTTGGTACCGTTTAAACAGGATGGAGACGAACTCCTCG primers were used to create the NP deletion 267–738 aa mutant. NP deletion mutant 402–738 aa was generated with forward ACGGGCCCTCTAGACTCGAGCGGCCGCGATCGCCATGGATTCTCGTCC and reverse TTTAAACTTAAGCTTGGTACCGTTTAAACCTCTTTTCTTAGAGTTACC primers. NP deletion mutant 534–738 aa was produced with forward ACGGGCCCTCTAGACTCGAGCGGCCGCGATCGCCATGGATTCTCGTCC and reverse TTTAAACTTAAGCTTGGTACCGTTTAAACAGGGCCTGGGACATTTTGAATTGG primers.

NP deletion mutant 667–738 aa was produced with forward ACGGGCCCTCTAGACTCGAGCGGCCGCGATCGCCATGGATTCTCGTCC and reverse TTTAAACTTAAGCTTGGTACCGTTTAAACCAAAACAGCATCAAATGGCCCCTG primers. Subsequently, NP deletion mutant 1–133 aa was produced with forward ACGGGCCCTCTAGACTCGAGCGGCCGCGATCGCCATGCTTGCTGCCATGCCGGAAGAGG and reverse TTTAAACTTAAGCTTGGTACCGTTTAAACCTGATGATGTTGC primers.

All described above pF4A CMV entry vectors were linearized with SgfII and PmeI restriction enzymes. Further cloning to LgBiT and SmBiT was done by enzymatic exchange with the entry pF4A CMV Flexi vectors carrying DNA fragments of all tested proteins. As a result, we obtained vectors expressing all tested proteins fused to N and C termini of both LgBit and SmBit. Their 8 combinations were tested in NanoBiT experiments to detect the optimal combination pair.

### NanoBiT assay

HEK293T cells were maintained in Dulbecco’s modified Eagle’s medium supplemented with 10% fetal bovine serum and 1% penicillin and streptomycin antibiotic solution. Cells were plated in 96-well white/clear bottom culture plates with 40% confluence and transient transfection was performed with the indicated constructs (1:1 ratio of interacting pairs) using Lipofectamine 3000 Plus (Invitrogen) in OPTI-MEM according to the manufacturers’ instructions. Twenty-four hrs post transfection, cells were treated with serial concentrations (1.3–14 μM) of 1E7–03 for an additional 6 hr. Nano-Glo Live Cell Substrate (N2012, Promega) was added and luminescence was measured using a GloMax-Multi Detection System (Promega). All experiments were performed at least three times.

### Western Blotting

To test NanoBiT construct expression, cells were lysed in whole cell lysis buffer (50 mM Tris-HCl, pH 7.5, 0.5 M NaCl, 1% NP-40, and 0.1% SDS) supplemented with protease and phosphatase cocktail and separated on a 10% polyacrylamide gel, transferred to PVDF membranes (Millipore, Allen, TX).Protein bands were detected with indicated NP (IBT Bioservices) and PP1 (Upstate) antibodies using horseradish peroxidase-linked secondary antibodies.

### Co-immunoprecipitation

GFP tagged NP-WT and NP E619K vectors were co-transfected with Flag- or V5-tagged PP1α in 293T cells. Twenty-four hrs post-transfection, the cells were treated with DMSO or 1E7–03 (10 μM) for overnight. The protein lysate was prepared using a whole cell lysis buffer supplemented with a protease and phosphatase cocktail. Protein extract (250 μg) was supplemented with 1.5 μg of anti-FLAG (Sigma) or 2.0 μg of anti-V5 (Invitrogen) antibodies and incubated with pre-blocked protein A/G-agarose beads in 5% bovine serum albumin in TNN buffer (50 mM Tris-HCl, pH 7.5, 0.5% NP-40, 150 mM NaCl) for 4 hrs with rocking. After washing with TNN buffer, proteins precipitated with the agarose beads were resolved on 10% Bis-Tris SDS-PAGE, transferred to PVDF membrane, and probed with indicated antibodies.

### DSP cross-linking

293T cells were transfected with the WT NP or NP E619K expressing plasmids. Twenty-four hrs post transfection, the cells were treated with DMSO or 1E7–03 (10 μM) for overnight. Then, the cells were incubated with 2 mM dithiobis(succinimidyl propionate) DSP cross-linker (Thermo Scientific) for 45 min at room temperature. The cells were then lysed in the modified whole cell lysis buffer that was devoid of SDS, resuspended in a non-reducing sample buffer, and subjected to SDS-PAGE and immunoblot analysis.

### Transmission electron microscopy (TEM)

To analyze the NP-mediated capsid formation, 293 cells were transfected with vectors expressing NP-mCherry (WT or E619K mutant) alone or in combination with VP24 and VP35 expressing vectors. The cells were additionally treated with 3 μM 1E7–03 starting at 18 hrs after the transfection. At 48 hrs post transfection, the cells were incubated in a warm fixative solution (120 mM Sodium Cacodylate pH 7.4, 2.5% glutaraldehyde, 1% paraformaldehyde) for 20 min at room temperature and stored at 4°C overnight. Cells were then osmicated by incubating 120 mM Sodium Cacodylate pH 7.4 supplemented with 1% OsO_4_ for 1 hr and then overnight in water solution of 1% Uranyl Acetate at 4°C. The cells were then dehydrated through the series of EtOH dilutions from 30–100%, embedded in LX112 epoxy resin and heated at 60°C for 48 hrs. Sample blocks were sectioned *on face* and post-stained with 1% uranyl acetate in water and Reynold’s lead. All imaging was performed at 80 KV in a Talos 200X transmission electron microscope (TEM) (Thermo Fisher Scientific).

### Fluorescent microscopy

To analyze the NP interaction with PP1, 293T cells were transfected with vectors expressing NP-mCherry in combination of PP1α-eGFP, PP1β/δ-eGFP or PP1γ-eGFP [42]. At twenty-four hrs post transfection, the cells were treated with Hoechst and photographed on Olympus IX73 (Olympus, Center Valley, PA) using filters for DAPI, Texas Red and FITC fluorescence with 600X magnification. Colocalization was quantified using Manders coefficient which is defined as MOC=∑i(Ri×Gi)/√(∑iR2i×∑iG2i) where Ri and Gi are the average level of grey from the red and green fluorescence respectively [43]. Manders coefficient was calculated in ImageJ using the JACoP plug-in.

### Flow cytometry analysis

The 293 cells were co-transfected with either NP-mCherry, VP24 and VP35 (3:1:1 ratio) plasmids or with NP E619K-mCherry, VP24 and VP35 plasmids using Lipofectamine Plus reagent (Thermo Fisher Scientific). Transfected cells were treated with either 1E7–03 (1 μM) or DMSO for overnight. Cells were trypsinized, washed with 1 ml of PBS, resuspended in 0.5 ml of PBS, and analyzed using FACSVerse (Becton Dickinson, Franklin Lakes, NJ). Flow cytometry analysis was conducted in triplicates.

### NP molecular presentation

The previously described de novo NP structure [31] was used to prepare NP images in Chimera 1.14 (https://www.cgl.ucsf.edu/chimera).

### Statistical analysis

All graphs were prepared using GraphPad Prism 6 software. The data were presented as mean ± SD or standard error of the mean (SEM) as indicated in the figure legends. Statistical comparison was done with Student’s *t* test. Where indicated, non-linear regression analysis was performed to determine IC_50_ using GraphPad Prism 6 built-in algorithms.

## Data Availability

All vectors generated during this study and original TEM images are available upon request from corresponding authors.
